# Correction to “Design of Tau Aggregation Inhibitors Using Iterative Machine Learning and a Polymorph-Specific Brain-Seeded Fibril Amplification Assay”

**DOI:** 10.1021/jacs.5c18723

**Published:** 2026-03-09

**Authors:** Alessia Santambrogio, Robert I. Horne, Michael A. Metrick, Nicholas C. T. Gallagher, Z. Faidon Brotzakis, Dillon Rinauro, Ergina Vourkou, Katerina Papanikolopoulou, Efthimios M. C. Skoulakis, Sara Linse, Byron Caughey, Michele Vendruscolo

The following should be added to the Acknowledgments:

K.P. and E.M.C.S. acknowledge funding from the Hellenic General Secretariat for Research and Innovation (TAEDR-0535850) through the National Research Network for highlighting the genetic basis of the neurodegenerative Alzheimer’s and Parkinson’s diseases, the detection of reliable biomarkers, and the development of innovative computational technologies and therapeutic strategies based on precision medicine.

