# Supplementary material for: Design of Tau Aggregation Inhibitors Using Iterative Machine Learning and a Polymorph-Specific Brain-Seeded Fibril Amplification Assay
Source: J Am Chem Soc. 2025 Sep 18;147(39):35942–52. doi: 10.1021/jacs.5c12812 (PMC12498416; doi:10.1021/jacs.5c12812)
Supplement: Supplementary file 1 [file ja5c12812_si_001.pdf]

SUPPLEMENTARY INFORMATION

Design of Tau Aggregation Inhibitors Using Iterative Machine Learning and a Polymorph-Specific Brain-Seeded Fibril Amplification Assay

Alessia Santambrogio[1,+], Robert I. Horne[1,+], Michael A. Metrick II[1,2,3,+], Nicholas C. T. Gallagher[1], Z. Faidon Brotzakis[1,6], Dillon Rinauro[1], Ergina Vourkou[4], Katerina Papanikolopoulou[4], Efthimios M. C. Skoulakis[4], Sara Linse[5], Byron Caughey[2], Michele Vendruscolo[1,*]

[1]*Centre for Misfolding Diseases, Yusuf Hamied Department of Chemistry, University of Cambridge, Cambridge CB2 1EW, UK*
[2]*Laboratory of Neurological Infections and Immunity, National Institute for Allergy and Infectious Diseases, Hamilton, MT 59840, USA*
[3]*Departments of Pathology and Laboratory Medicine, University of California at San Francisco, CA 94143, USA*
[4]*Institute for Fundamental Biomedical Research, Biomedical Sciences Research Centre "Alexander Fleming", Vari 16672, Greece*
[5]*Biochemistry and structural biology, Lund University, 22100 Lund, Sweden*
[6]*Institute for Bioinovation, Biomedical Sciences Research Centre "Alexander Fleming", Vari 16672, Greece*

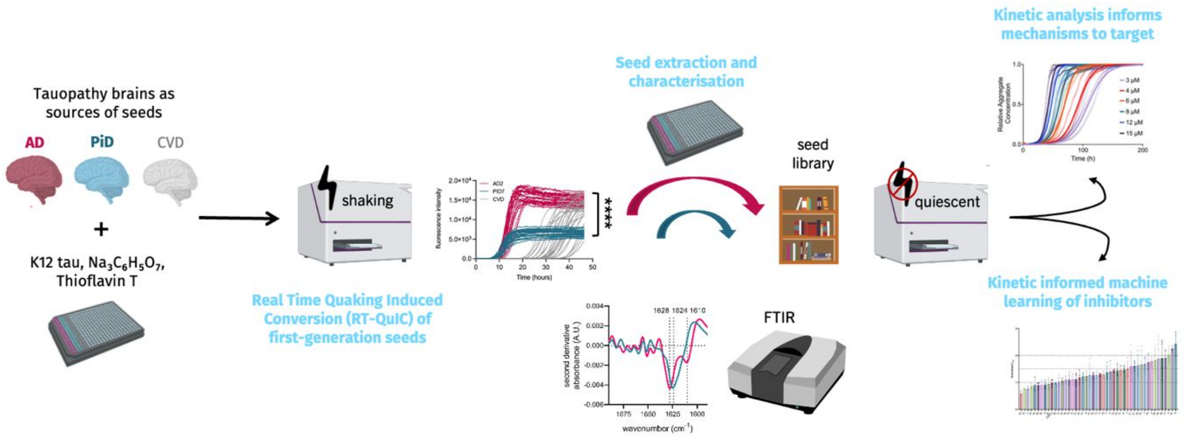

Figure S1. **Schematic illustration of the protocol for polymorph-specific drug design assay used in this work**. Brain-derived seeds are obtained from tauopathy and control brain homogenates and used to promote the *in vitro* conversion of recombinant K12 tau into aggregates. Recombinant seeds are then extracted from this aggregation reaction to form libraries for downstream biophysical characterization and use in drug design assays. In these assays, recombinant tau monomers are reacted with recombinant seed libraries in the presence of docked compounds over rounds of iterative machine learning, informed by kinetic analysis of inhibition. This approach yields potent polymorph-specific tau aggregation inhibitors. (AD, Alzheimer’s disease; PiD, Pick’s disease; CVD, cerebrovascular disease).

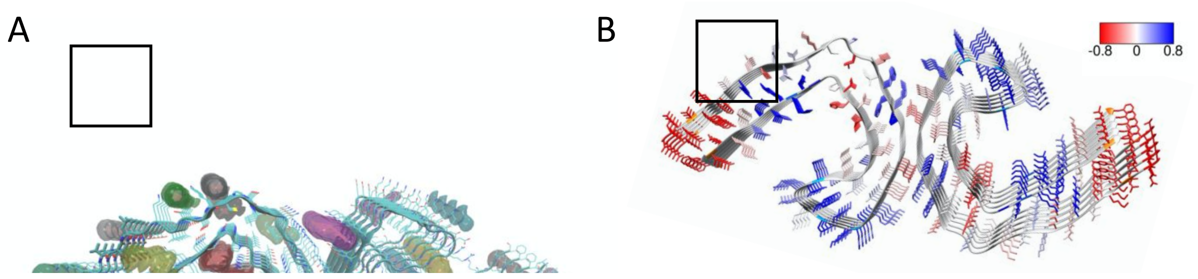

Figure S2. Binding site predictions based on pocket identification and solubility analysis. (A) Cavity-based binding pocket prediction using Fpocket[1]. **(B)** Solubility-based scoring of the predicted binding pockets using CamSol[2]. The black box indicates residues 313-315, where both solubility is low and cavity propensity is high. This approach was recently validated for the structure-based discovery of α-synuclein aggregation inhibitors[3].

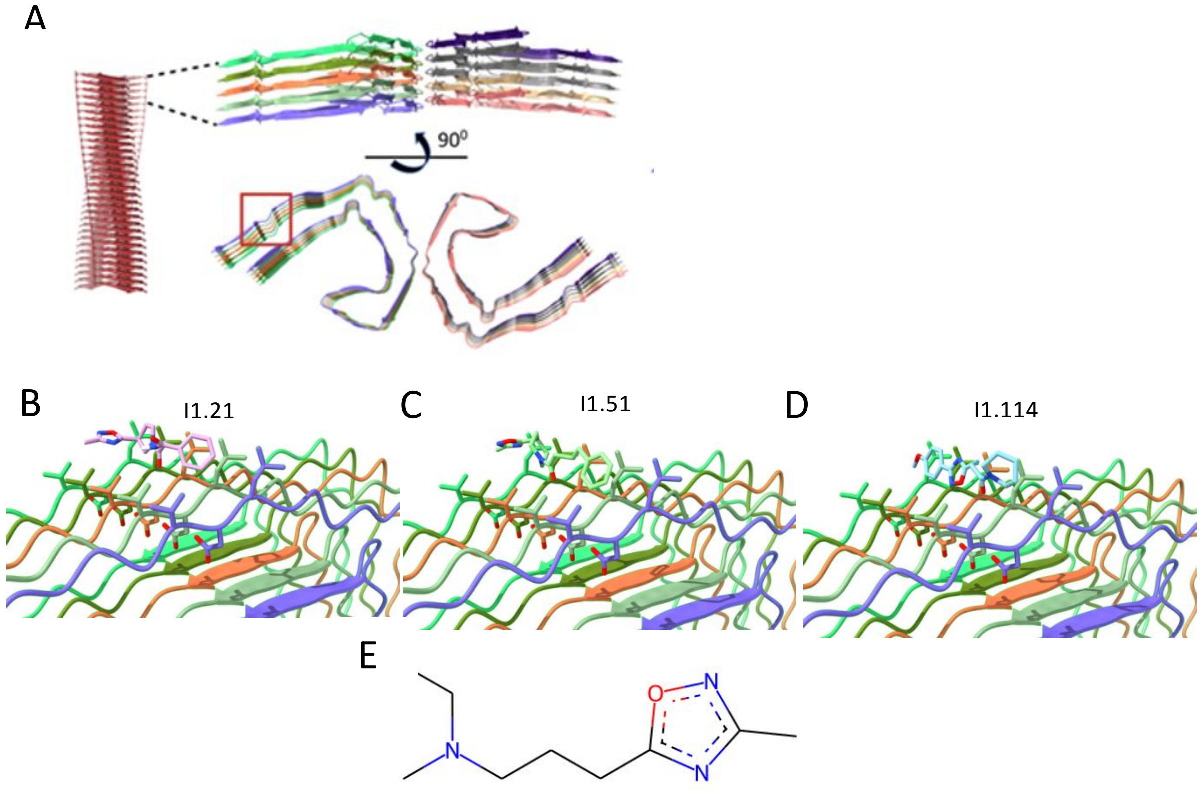

Figure S3. **Small molecule features**. (**A**) Schematic of the proposed binding mechanism, showing the cross section of the *ex vivo* AD tau fibril structure[4] and the targeted binding site. Binding of I1.21 in this pocket as predicted by AutoDock Vina is also shown. (**B-D**) Best docking poses for the three compounds (I1.21 (B), I1.51 (C), and I1.114 (C)) against the AD tau fibril (PDB:5O3L) at the binding site (V313-L315). (**E**) Maximum common substructure between compounds, determined by RDKit.

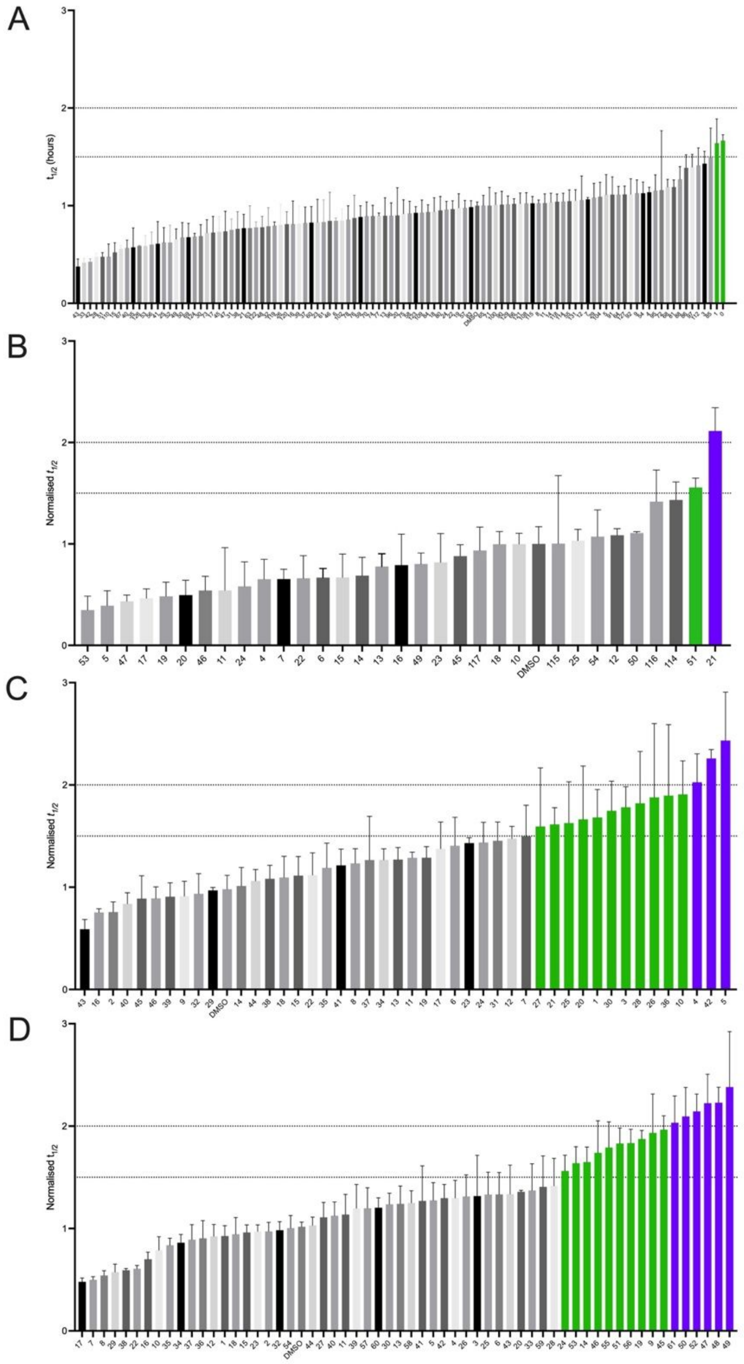

Figure S4. Summary of the aggregation data for the compounds tested in this work in the presence of first-generation fibrils. Bars represent the average half time ($t_{1/2}$) of 4 replicate reactions of 5 µM K12 tau monomer, 50 nM first-generation AD-seeded K12 fibrils in the presence of 1% DMSO (error=SD) and 20 µM molecules I1.21, I1.51 and I1.114; averages are normalised to DMSO = 1. Green bars represent reactions with $t_{1/2}$ > 1.5 greater or more than DMSO control, indicating aggregation inhibitors. Purple bars represent reactions with greater than two-fold $t_{1/2}$ prolongation. **(A)** $t_{1/2}$ values of AD-seeded K12 tau in the presence of original docked compounds. **(B)** $t_{1/2}$ values of the same reactions in the presence of compounds from first iteration of kinetic-informed machine learning. **(C)** As above for iteration two. **(D)** As above for iteration three.

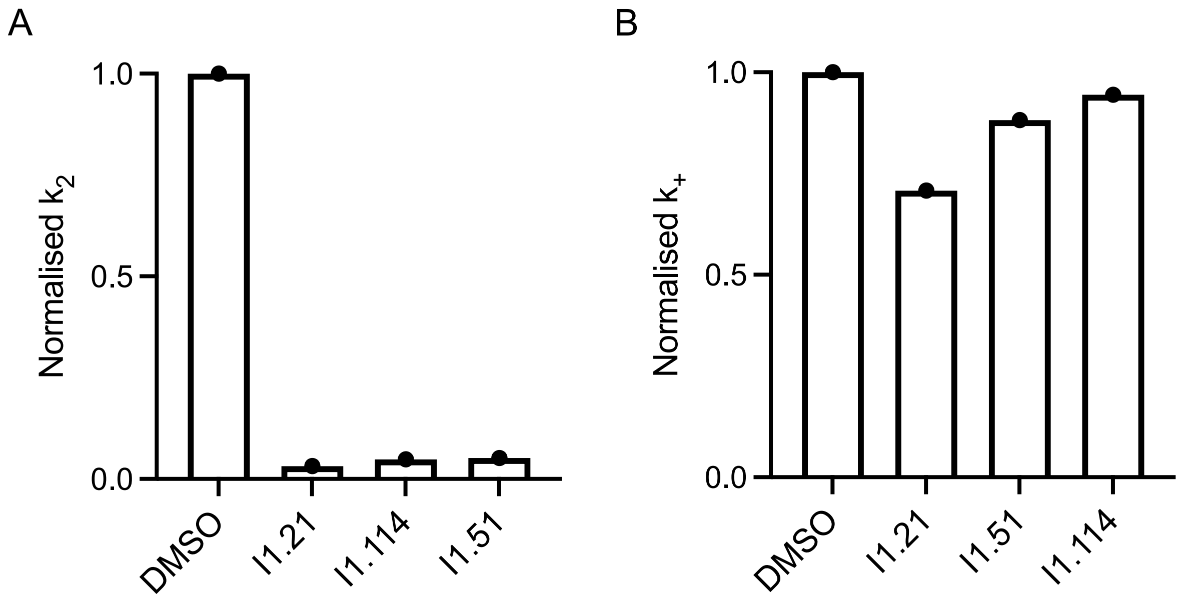

Figure S5. Secondary nucleation rate constant (k_2) and elongation rate constat (k_+) for the molecules tested in this work. k_2 (**A**, green) and k_+ (**B**, teal) values obtained when fitted aggregation curves in **Figure 4B** to a secondary nucleation model at 20 μM (4:1 stoichiometry) of I1.21, I1.51 and I1.114.

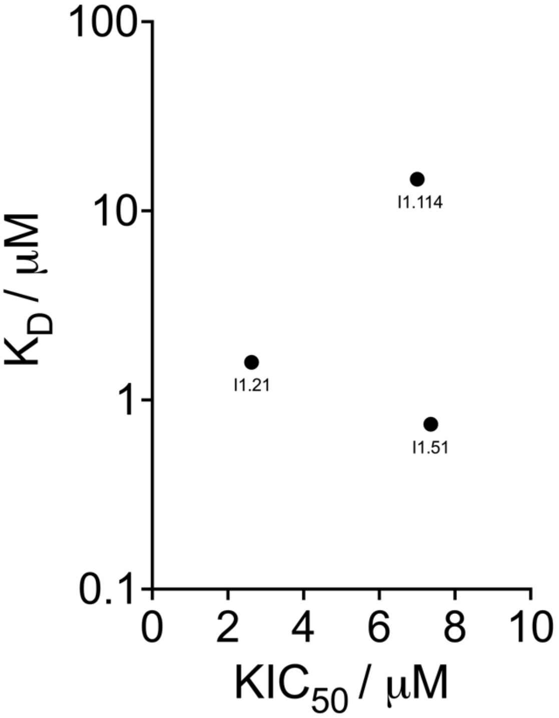

Figure S6. Comparison of the KIC_{50} and K_D values for the 3 small molecules reported in this work. The K_D values report on the binding affinity of the small molecules for the tau fibrils, while the KIC_{50} values quantify their potency as inhibitors of fibril proliferation.

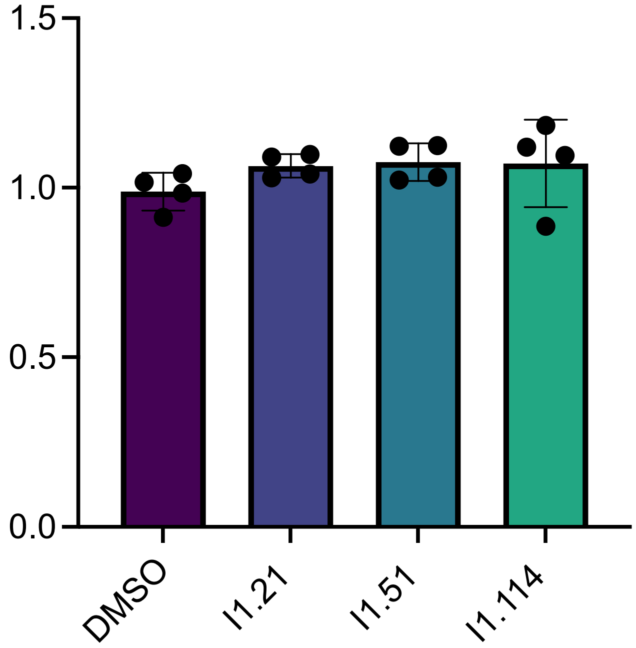

Figure S7. PiD brain homongenate-seeded K12 aggregtion $t_{1/2}$ in the presence of the 3 small molecules reported in this work. Normalised half-time ($t_{1/2}$) of PiD-seeded K12 reactions for the most potent AD-seeded K12 aggregation inhibitors described in this work.

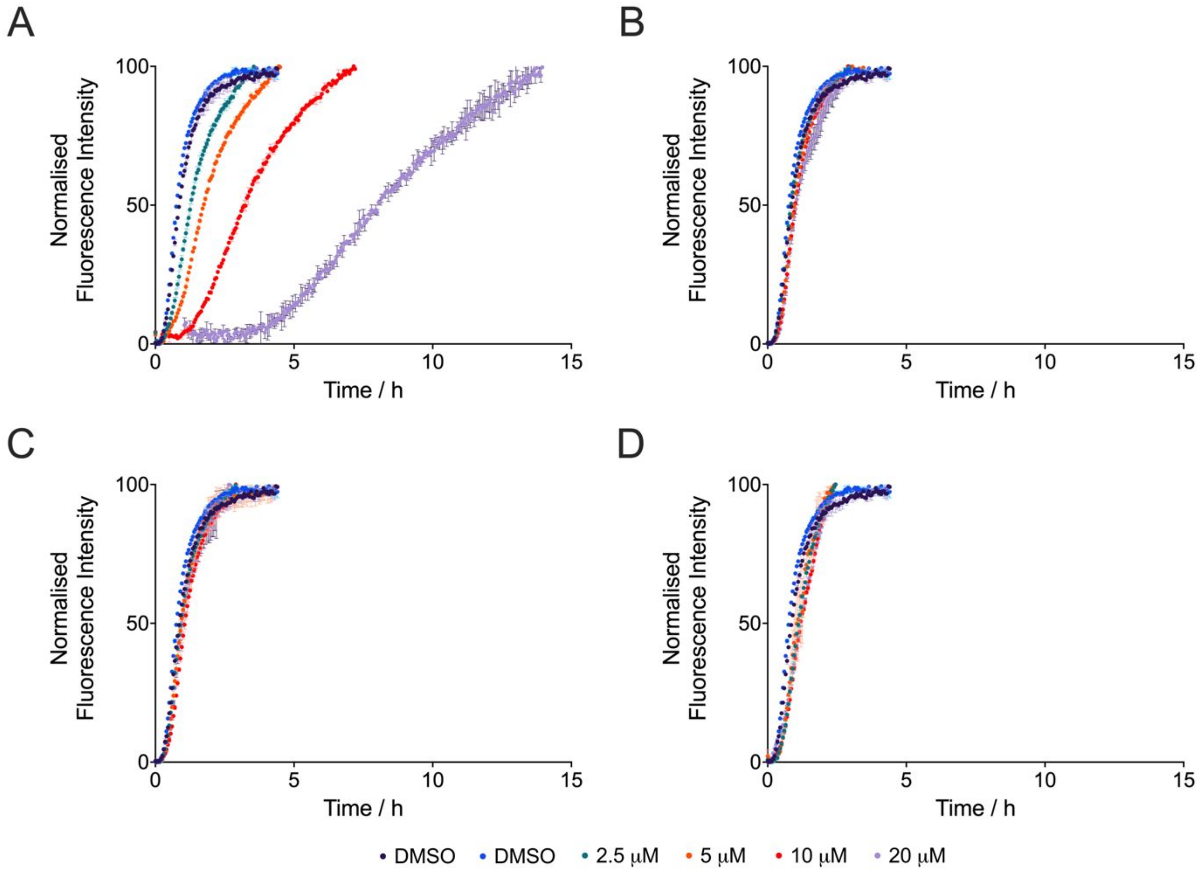

Figure S8. Kinetic traces of Aß42 aggregation in the presence of the 3 small molecules reported in this work. Aß42 (40 nM seed, 2 μM monomer) was aggregated in the presence of 1% DMSO (purple and blue points), and molecules at 2.5 μM (teal), 5 μM (orange), 10 μM (red), and 20 μM (lilac). (**A**) Adapalene, a positive control with previously reported anti-aggregation potency[5]. (**B**) Compoud I1.21. (**C**) Compoud I1.51. (**D**) Compoud I1.114.

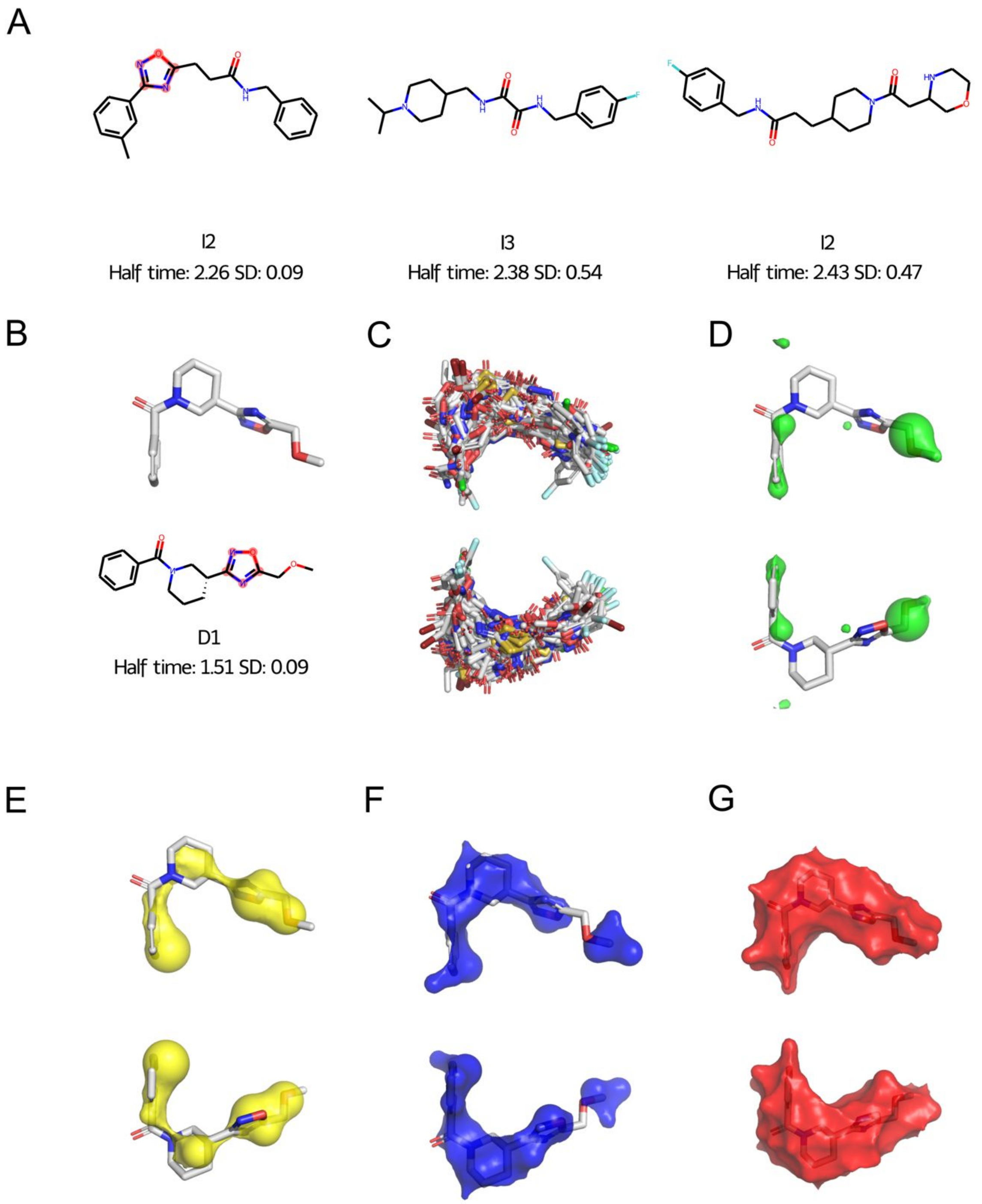

Figure S9. Pharmacophore analysis for the molecule set tested in iterations 1-3. (A) 2D structures of the top 3 aggregation inhibitors, highlighting the recurrent oxadiazole moiety where present. Activities are indicated below each structure. **(B)** 3D and 2D structure of d1, the first molecule identified which contained the oxadiazole moiety (highlighted). All other structures were aligned to this. Activity is indicated below the structure. **(C)** 3D conformers of the screening molecules overlapped and aligned (rotated around the x-axis from top to bottom). **(D)** Aromatic ring system prevalence (green), **(E)** Hydrophobic prevalence (yellow), **(F)** H bond donor prevalence (blue), **(G)** H bond acceptor prevalence (red) all weighted by the screening experimental half-time.

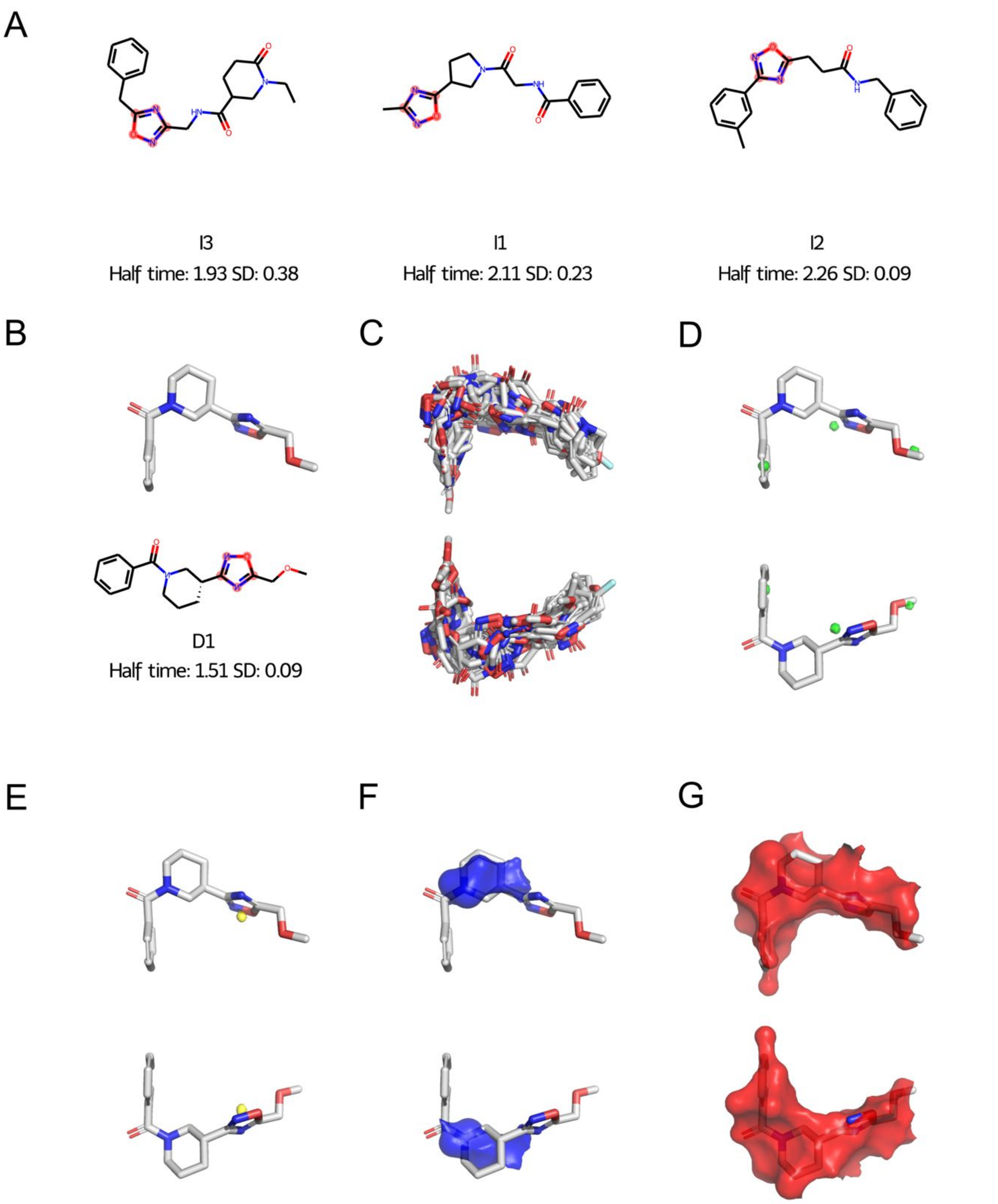

Figure S10. Pharmacophore analysis for the oxadiazole set tested in iterations 1-3. (A) 2D structures of the top 3 aggregation inhibitors, highlighting the oxadiazole moiety. Activities are indicated below each structure. **(B)** 3D and 2D structure of d1, the first molecule identified which contained the oxadiazole moiety (highlighted). All other structures were aligned to this. Activity is indicated below the structure. **(C)** 3D conformers of the screening molecules overlapped and aligned (rotated around the x-axis from top to bottom). **(D)** Aromatic ring system prevalence (green), **(E)** hydrophobic prevalence (yellow), **(F)** H bond donor prevalence (blue), **(G)** H bond acceptor prevalence (red) all weighted by the screening experimental half-time.

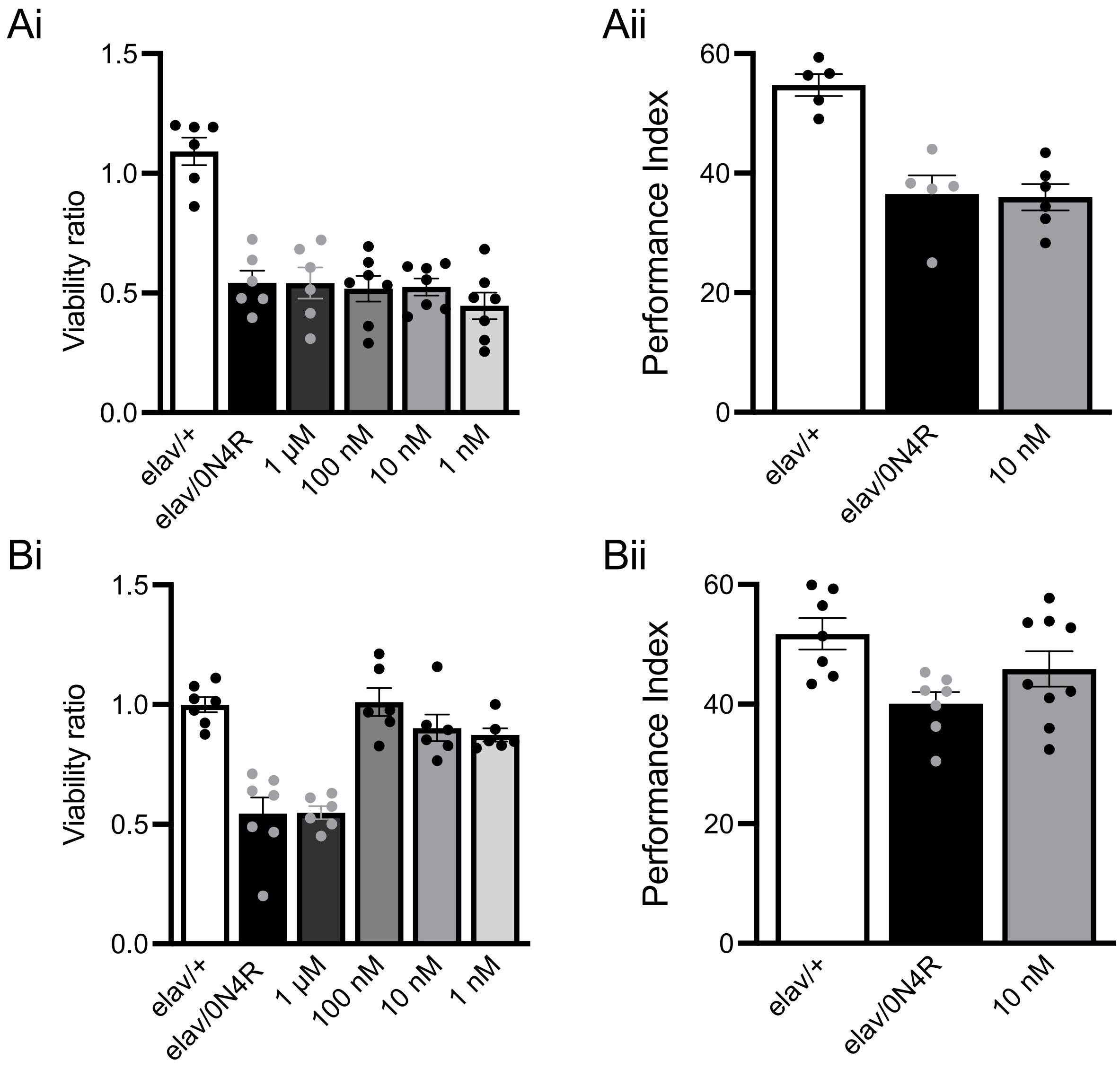

Figure S11. Effects of two polymorph-specific tau aggregation inhibitors (I1.51 and I.114) on flies expressing human 0N4R tau. **(Ai)** Viability of flies treated with vehicle (control) and increasing concentrations of compound I1.51. (**Aii**) Twenty-four-hour Spaced Conditioning memory (PSD-M) performance of flies with or without 10 µM of compound I1.51. **(Bi)** As above for I1.114. **(Bii)** As above for I1.114. For all the experiments bars indicate mean ± SEM and and stars (*) indicate significant differences from both controls (elav/+). Statistical details are presented in Supplementary **Table S2** and **S3**.

Table S1. Molecule metrics. (A) Global rate constants for protein only kinetics in Figure 2. **(B)** Normalised rate constants for light seeded dose dependence of molecules I1.21, I1.51 and I1.114. **(C)** Binding constants of the same molecules to tau fibrils, and kinetic inhibitory constant (KIC_{50}) values, the molecule concentration at which the half time of aggregation of the light seeded assay is extended by 50%.

A

Sample	Rate constant	
	k_2 (M^{-2} h^{-1})	k_p (M^{-1} h^{-1})
Fig. 2 data	3512190116	95166

B

Sample	Normalized rate constant	
	k_2	k_p
0 μM	1	1
21: 20 μM	0.03	0.71
21: 10 μM	0.14	0.71
21: 5 μM	0.42	0.81
51: 20 μM	0.05	0.94
51: 10 μM	0.39	0.94
51: 5 μM	1.16	1.06
114: 20 μM	0.05	0.88
114: 10 μM	0.27	0.88
114: 5 μM	0.96	0.94

C

Molecule	Equilibrium constants	
	Binding constant (K_D) / μM ± SD	Kinetic inhibitory constant (KIC_{50}) / μM ± SD
21	1.58 ± 0.15	2.6 ± 0.5
51	0.74 ± 0.28	7.4 ± 5.0
114	12.50 ± 9.6	7.0 ± 3.6

Table S2: Comparisons to control elav^{C155}-Gal4> w^{1118} using Dunnett's test.

**d I1.21**	**p-value**	**I1.51**	**p-value**	**I1.114**	**p-value**
elav/+	1	elav/+	1	elav/+	1
elav/0N4R	1e-07	elav/0N4R	2e-07	elav/0N4R	3e07
tau 100 nM	1	tau 1 μM	2e-07	tau 100 nM	1
tau 10 nM	1	tau 10 nM	4e-08	tau 10 nM	0.5
tau 250 nM	1e-05	tau 100 nM	3e-08	tau 1 nM	0.2
tau 1 nM	2e-09	tau 1 nM	0	tau 1 μM	7e-07

Table S3: Comparisons to control elav^{C155}-Gal4> w^{1118} and elav^{C155}-Gal4> 0N4R using LSM.

Genotype	Mean	± SEM	p-value	F-ratio
**LTM 10nM d21**	**ANOVA $F_{(2.23)}$=18 p=0.00003**			
elav^{C155}-Gal4> w^{1118}	50	±2		
elav^{C155}-Gal4 >0N4R	37	±1	**6e-06**	36
elav^{C155}-Gal4 >0N4R I1.21	44	±2	**0.01**	7
elav^{C155}-Gal4> 0N4R	37	±1		
elav^{C155}-Gal4 >0N4R I1.21	44	±2	**0.003**	11
**LTM 10nM d51**	**ANOVA $F_{(2.15)}$=18 p=0.0002**			
elav^{C155}-Gal4> w^{1118}	56	±3		
elav^{C155}-Gal4 >0N4R	37	±3	**0.0002**	25
elav^{C155}-Gal4 >0N4R I1.51	36	±2	**0.0001**	29
elav^{C155}-Gal4> 0N4R	37	±3		
elav^{C155}-Gal4 >0N4R I1.51	36	±2	0.9	0.02
**LTM 10nM d114**	**ANOVA $F_{(2.22)}$=4 p=0.03**			
elav^{C155}-Gal4> w^{1118}	52	±3		
elav^{C155}-Gal4 >0N4R	40	±2	**0.008**	9
elav^{C155}-Gal4 >0N4R I1.114	46	±3	0.1	3
elav^{C155}-Gal4> 0N4R	40	±2		
elav^{C155}-Gal4 >0N4R I1.114	46	±3	0.1	2

Supplementary References

1. Le Guilloux, V.; Schmidtke, P.; Tuffery, P., Fpocket: an open source platform for ligand pocket detection. *BMC Bioinformatics* **2009,** *10*, 1-11.
2. Sormanni, P.; Aprile, F. A.; Vendruscolo, M., The CamSol method of rational design of protein mutants with enhanced solubility. *J. Mol. Biol.* **2015,** *427* (2), 478-490.
3. Horne, R. I.; Andrzejewska, E. A.; Alam, P.; Brotzakis, Z. F.; Srivastava, A.; Aubert, A.; Nowinska, M.; Gregory, R. C.; Staats, R.; Possenti, A., Discovery of potent inhibitors of α-synuclein aggregation using structure-based iterative learning. *Nat. Chem. Biol.* **2024,** *20*, 634-645.
4. Fitzpatrick, A. W.; Falcon, B.; He, S.; Murzin, A. G.; Murshudov, G.; Garringer, H. J.; Crowther, R. A.; Ghetti, B.; Goedert, M.; Scheres, S. H., Cryo-EM structures of tau filaments from Alzheimer's disease. *Nature* **2017,** *547* (7662), 185-190.
5. Habchi, J.; Chia, S.; Limbocker, R.; Mannini, B.; Ahn, M.; Perni, M.; Hansson, O.; Arosio, P.; Kumita, J. R.; Challa, P. K., Systematic development of small molecules to inhibit specific microscopic steps of Aβ42 aggregation in Alzheimer's disease. *Proc. Natl. Acad. Sci. USA* **2017,** *114* (2), E200-E208.
